# Schizophrenia spectrum stigma in healthcare: a systematic review

**DOI:** 10.3389/fpsyt.2025.1648957

**Published:** 2025-08-25

**Authors:** Elizabeth A. Kruse, David Dodell-Feder

**Affiliations:** ^1^ Department of Psychology, University of Rochester, Rochester, NY, United States; ^2^ Department of Neuroscience, University of Rochester Medical Center, Rochester, NY, United States

**Keywords:** schizophrenia, stigma, healthcare, providers, bias

## Abstract

**Introduction:**

Within healthcare settings, schizophrenia spectrum disorder (SSD) stigma is pervasive and presents significant barriers to recovery and equitable care. Understanding the sources, nature, and moderators of such stigma among healthcare providers is essential for informing targeted interventions.

**Methods:**

We conducted a systematic review of 44 peer-reviewed studies examining SSD-related stigma among diverse healthcare providers, including trainees, nurses, general practitioners, psychiatrists, psychologists, and community health workers. Studies were synthesized to identify common manifestations of stigma, as well as professional and demographic moderators.

**Results:**

Stigma was observed across all healthcare professions, manifesting through stereotypes, prejudices, and discriminatory behaviors. Consistent patterns included higher stigma among generalist providers compared to mental health specialists and reduced stigma associated with greater professional experience and personal contact with individuals with SSDs. Findings regarding other moderators, such as age, gender, and etiological beliefs, were mixed.

**Discussion:**

SSD stigma is widespread within healthcare and varies by provider type, training, and personal experience. Interventions to reduce stigma should be tailored to specific provider groups and contexts. Future research should employ longitudinal and mixed-method designs to clarify moderator effects and assess targeted anti-stigma strategies.

## Introduction

Schizophrenia spectrum disorders (SSDs) are among the most stigmatized mental health disorders in the world (1–3). Affecting approximately 24 million people worldwide, SSDs represent a significant public health concern, contributing to chronic disability, reduced quality of life, and reduced life expectancy (4). The burden of disease is widespread across systems and resources, but, has the most severe impact on the affected individual, largely due to stigma—the social devaluation or disapproval of a person based on a perceived characteristic (5). Stigma among individuals with SSDs has been consistently associated with increased suicidal ideation and attempts, hopelessness and depression, and decreased quality of life, self-esteem, and social functioning (6–9). Its effects are so profound that it has been described as a “second illness” (10).

As recognition of SSD-stigma has grown, efforts have been made to understand how it forms and persists. Stigma has several dimensions: perceived stigma (belief that others hold negative views), anticipated stigma (expectation of future discrimination), experienced stigma (actual discriminatory encounters), and self-stigma (the internalization of stigma leading to a negative self-view or “illness identity”; 11). These dimensions create a multifaceted barrier to recovery, influencing individuals’ willingness to seek help, adhere to treatment, and engage with social and professional networks. Stigma manifests through stereotypes (e.g., beliefs about dangerousness or incompetence), prejudices (e.g., negative evaluations involving fear or pity), and discriminatory behaviors (e.g., avoidance or exclusion) (12–14).

The stigma surrounding SSDs has deep historical roots, shaped by centuries of fear and misunderstanding. Early conceptions portrayed individuals as dangerous and morally deficient, often invoking supernatural explanations used to justify inhumane institutionalization of affected individuals (15–17). Though contemporary views and practices have evolved, harmful attitudes persist, reinforced by cultural narratives and media depictions that associate psychosis with violence (10). Stigma may be ubiquitous, but may be particularly consequential when held by healthcare providers, who often serve as the first point of contact and are well-positioned to influence the quality and trajectory of care. Paradoxically, research increasingly shows that stigma is pervasive even within healthcare settings, manifesting as implicit biases, avoidance behaviors, and therapeutic pessimism (18). Alarmingly, these attitudes are not confined to general healthcare providers; even mental health professionals (MHPs)—those most intimately involved in supporting individuals with SSDs—may perpetuate stigma through subtle or overt behaviors, such as underestimating patients’ capabilities, adopting overly paternalistic approaches, or failing to examine their own biases (19).

This raises the question: does stigma differ across provider types in ways that meaningfully affect care? Unfortunately, studies rarely compare stigma *between* provider groups (e.g., psychologists versus nurses), leaving unclear how stigma manifests across professions (1, 3, 20, 21). Differences are likely, given variations in education, training, patient contact, and professional roles. For example, a provider working within a recovery-oriented framework that promotes self-efficacy, and who has observed positive patient outcomes over the years, may differ significantly from someone with little experience working with SSDs who takes a strictly biogenetic perspective. Understanding how stigma varies by provider group, and the factors that shape these differences, is critical for developing targeted, sustainable stigma-reduction efforts.

Theoretically, provider stigma can be understood through an integration of social and affective frameworks. The contact hypothesis posits that stigma may be reduced by positive, frequent interactions in equitable environments—those promoting equal status, cooperation (i.e., shared decision making), and institutional support (22). Indeed, providers who work closely with individuals with SSDs, such as psychiatrists and mental health nurses, often exhibit lower stigma (23). However, contact alone may be insufficient; without adequate training or support, challenging interactions can reinforce negative attitudes and stereotypes (24). Thus, providers lacking positive, frequent contact may be especially prone to higher stigma. Complementing the contact hypothesis, attribution theory (25) explains how casual beliefs influence emotional and behavioral responses. In clinical care, providers may attribute SSDs to internal or external, stable or unstable, and controllable or uncontrollable causes. These attributions influence emotional responses—such as pity, frustration, or fear—which then guide behavior. For example, providers who attribute SSDs to uncontrollable, biogenetic factors may experience emotions such as pity, which can reduce blame, but increase paternalistic attitudes and reduce empathy (26, 27). Conversely, when SSDs are attributed to personal failure, providers may experience anger or frustration, reinforcing avoidance or judgment. Intergroup emotion theory (28) adds a critical group-based lens, highlighting how emotional responses are shaped not only by individual appraisals but by perceived group membership. When individuals with SSDs are viewed as part of an “outgroup,” emotions like fear or disgust may intensify, particularly when tied to stereotypes of dangerousness or unpredictability, which can drive stigmatizing behaviors (29–31). These affective responses, shaped by both attributional and group-based logic, are central to understanding stigma in clinical settings. Providers who feel unprepared to manage complex symptoms may experience these emotions more intensely, increasing the risk of avoidance and disengagement.

**Figure 1:**
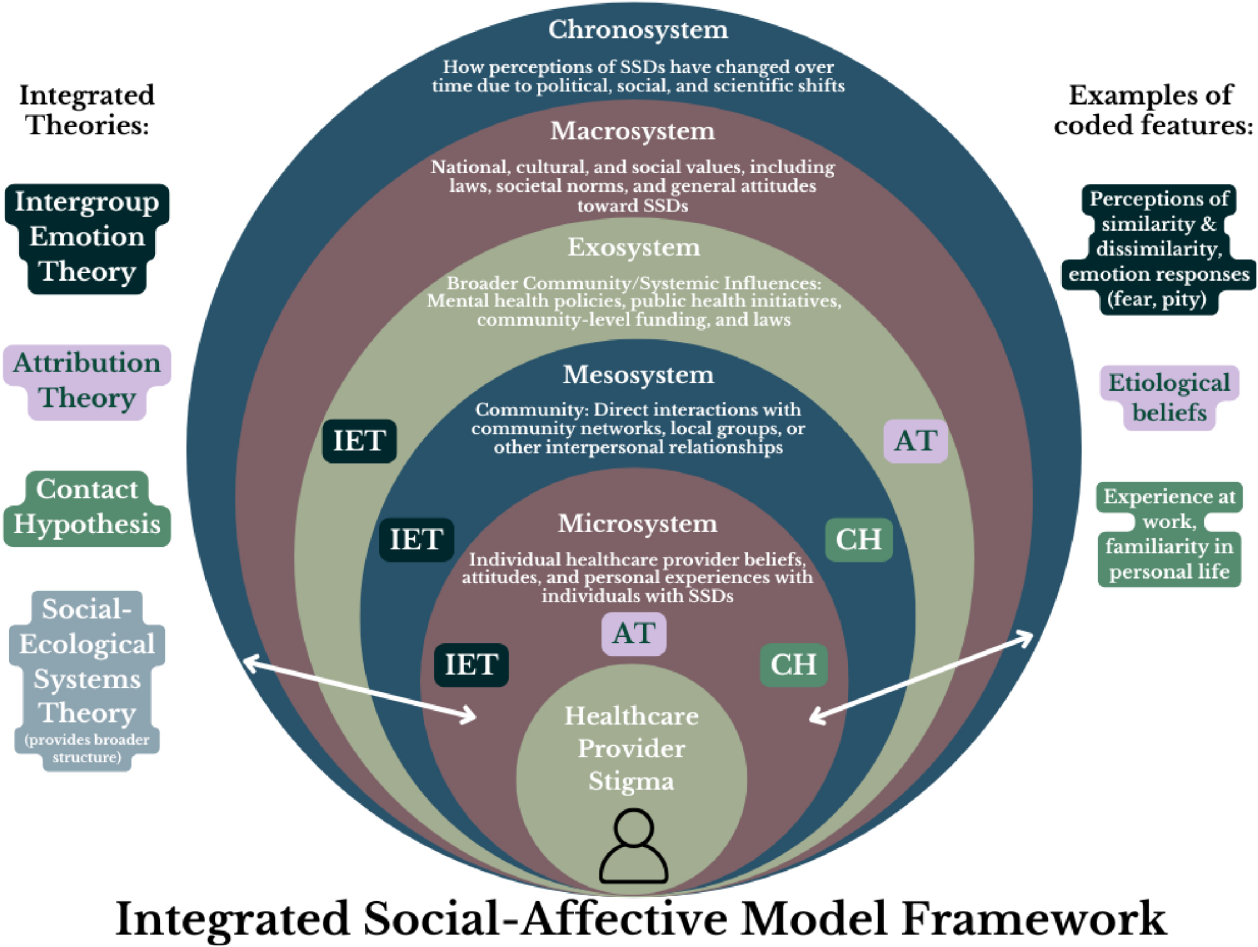
Visual depiction of an integrated social-affective model framework.

Adopting a broader lens, the social-ecological model (32) helps illustrate how stigma, and the aforementioned social and affective processes, operates across interdependent levels. Individually, providers may internalize societal narratives that portray SSDs as dangerous or untreatable. Interpersonally, these attitudes may manifest as microaggressions or therapeutic pessimism. Structurally, stigma can be embedded in institutions, a concept referred to as “structural stigma,” manifesting through factors such as underfunded services, insufficient training opportunities, or policy barriers that limit access to care (33). These conditions both reflect and reinforce stigma, shaping provider behavior and impeding equitable care (33). The social-ecological model highlights how stigma emerges through feedback loops, e.g., how individual provider beliefs (micro) shape institutional practices (macro), and how systemic factors reinforce individual attitudes. Within this framework, attribution and intergroup emotion theories explain how providers’ causal beliefs shape affective and behavioral responses, while the contact hypothesis highlights the role of experience. This integrated, multi-level model (**Figure 1**) offers a comprehensive account of how stigma develops and persists among healthcare providers, and underscores the need for interventions that target these intersecting levels.

Despite growing attention to provider-based stigma (2), significant gaps remain. Much of the literature focuses on subsets of providers, such as nurses (1), MHPs (2, 21, 34), or primary care providers (35), limiting generalizability and comparison across roles. Moreover, given widespread stigma toward psychotic symptoms (2, 36, 37), and the dimensional nature of psychosis (38, 39), it is important to examine stigma across the full schizophrenia spectrum. Thus, the aim of this systematic review was to synthesize research on stigma towards the full schizophrenia spectrum (i.e., psychotic-like experiences in the general population to a SSD) among different groups of healthcare providers. These insights are essential for designing effective, context-sensitive interventions.

## Methods

**Figure 2:**
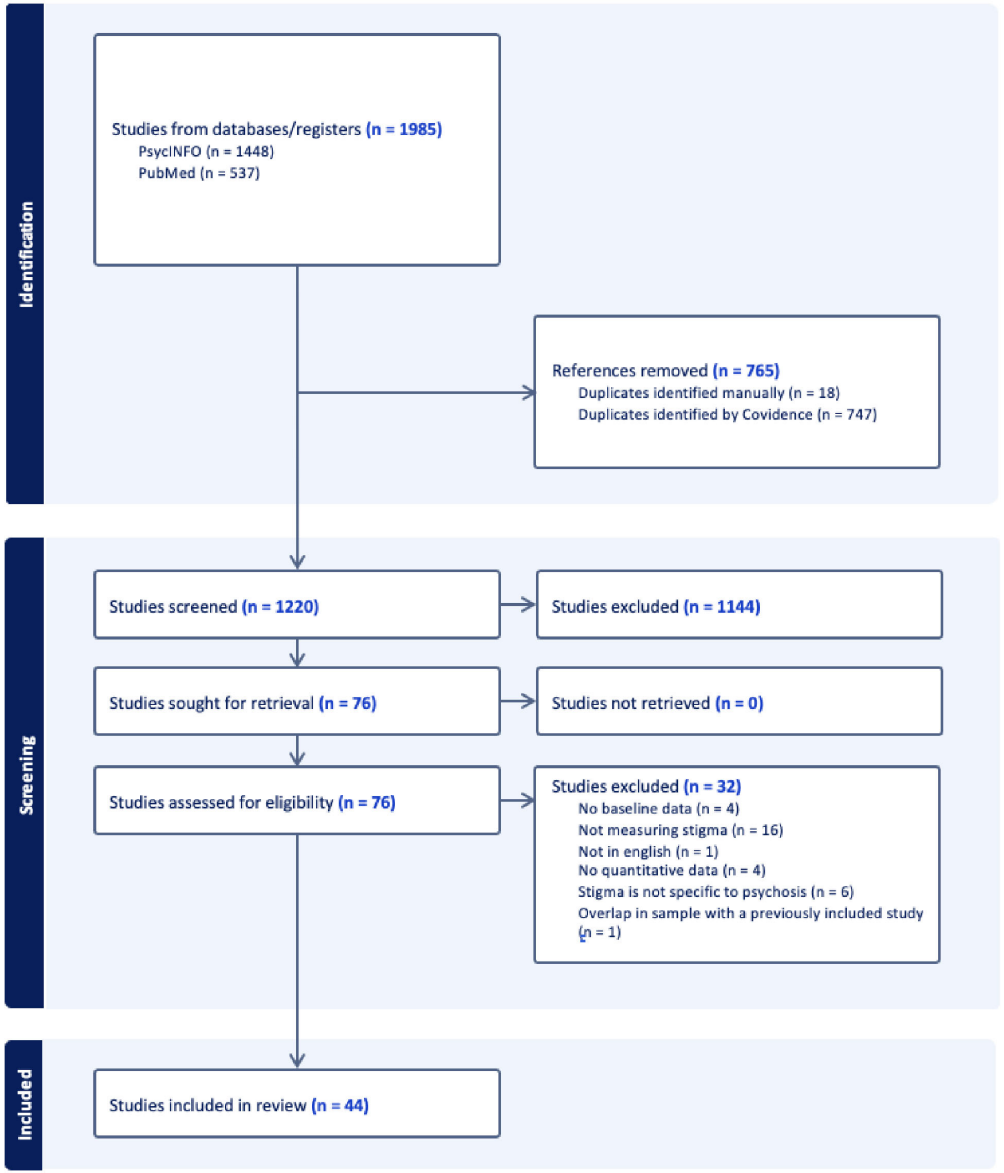
PRISMA flowchart for systematic search and identification of included studies.

This review adhered to the PRISMA 2020 guidelines (40), and the study selection process is outlined in the PRISMA flowchart (**Figure 2**). A review protocol was not pre-registered on PROSPERO. We did not conduct a formal assessment of the strength or certainty of evidence (e.g., using GRADE; 41) due to the narrative nature of the review, the heterogeneity of measures and outcomes across studies, and the primary aim of characterizing provider-based stigma rather than evaluating intervention effects.

### Eligibility criteria

Studies were included for review if they were: written in English; contained original data; were published in a peer-reviewed journal; collected and reported quantitative data; investigated stigma related to the schizophrenia spectrum specifically (versus mental illness broadly); and included participants that were healthcare providers. ​​We did not impose a publication date restriction in order to capture the full range of relevant literature on schizophrenia stigma among healthcare providers. However, we acknowledge that attitudes regarding SSDs have shifted over time, and we further address this consideration in the Limitations section.

### Search strategy

The literature search was conducted in two electronic databases—PsycINFO and PubMed—using search terms for schizophrenia/psychosis, stigma, and provider: (schizo* OR psychosis OR psychotic) AND (stigma OR attitude OR stereotype OR bias OR discrimination OR prejudice) AND (professional OR provider OR doctor OR medical provider OR healthcare provider OR mental health professional OR nurse OR psychologist OR psychiatrist OR therapist OR social worker OR case manager OR paramedic OR EMT). The initial search on September 16, 2024 had the following output: 633 records (PsycINFO), 491 records (PubMed). After initial pilot screening, to capture healthcare providers in training and “physicians,” we added the following terms on October 12, 2024: (OR medical student OR graduate student OR trainee OR student OR physician). The second search had the following unique output (i.e., after removal of duplicates from initial search): 797 records (PsycINFO), 10 records (PubMed). A final update search with all search terms was conducted on April 30, 2025, and had a date constraint from 09/16/2024 to 04/30/2025 to capture only newly published records. This search had the following output: 18 records (PsycINFO), 36 records (PubMed). The first author and an undergraduate research assistant performed the initial round of screening using the title and abstract. Next, full texts were retrieved, coded by the first author (see below), and evaluated against our inclusion criteria. Discrepancies in screening decisions were discussed, and consensus was reached through adjudication by both authors.

### Coding procedures

Study screening and coding were performed using Covidence, a web-based collaboration software platform that streamlines systematic reviews (42). In the initial screening phase, studies were excluded (*n *= 1102) if they were not original research (e.g., review papers, editorials, etc.), did not include healthcare providers as participants in their sample (e.g., measurement of experienced stigma from client perspective), did not mention stigma, did not mention SSDs, or were not written in English.

Study features were selected using a hybrid approach. Core coding categories (e.g., study design, sample characteristics, stigma measurement, the majority of the moderators) were determined *a priori* based on prior literature and systematic review guidelines (2, 43). However, additional features (e.g., moderator of personal experience with mental illness) emerged during the review and were incorporated into the coding framework as they became relevant. This approach ensured both theoretical rigor and responsiveness to the data.

The results are organized by key study features. First, findings are presented by provider type, the primary focus of this review. Next, results are organized by potential moderators of stigma (e.g., age, gender, workplace setting). Stigma features (e.g., social distance, perceptions of dangerousness) are highlighted throughout both sections. This structure is intended to systematically address the who, what, and why of stigma, capturing components within each aspect of the integrated social-affective model framework (**Figure 1**).

## Results

### Overview of results

*N* = 1985 records were identified through database searches, with 765 duplicates removed (**Figure 2**). After completing Title/Abstract screening and Full-Text screening, 44 articles were included in the review. The majority of articles removed during Full-Text screening were due to not measuring stigma (*n* = 16).

#### Study characteristics

The 44 studies reviewed span a wide range of countries (*N* = 21), reflecting diverse healthcare systems and cultural contexts (**Table 1**). The United States accounted for the largest percentage of the studies (20%, *n* = 9). Sample sizes varied considerably, ranging from small cohorts of fewer than 30 participants to larger studies exceeding 1,000 participants. The mean sample size across studies was 320 (*SD* = 470).

**Table 1:** Summary of studies investigating stigma in healthcare providers.

Author/Year (Country)	Participants	Diagnosis of Focus/Setting	Measure of stigma	Key findings
Ayano et al., 2017 (44) (Ethiopia)	Primary health care providers (*N* = 94; *n* = 44 health officers, *n* = 22 diploma nurses and *n* = 28 bachelor of science [BSC] nurses)	Psychosis/Primary Care	*Author-designed attitude questionnaire	Providers had the lowest knowledge (66% poor knowledge at baseline), lowest favorability ratings (66% unfavorable), and lowest percentage of identification through vignette for psychosis compared to three other diagnoses
Baba et al., 2017 (45) (Japan)	Psychiatrists, social workers, and psychologists (*N* = 119)	Psychotic-like-experiences (PLEs), at-risk mental state (ARMS), Schizophrenia/Medical Center	*21-item prejudice scale; Social Distance Scale (SDS)	Psychiatric providers exhibited the least prejudice and discrimination towards PLEs and ARMS compared to schizophrenia and depression, with prejudice emerging in early disease stages and discrimination increasing in later stages.
Bjorkman et al., 2008 (46) (Sweden)	Somatic and psychiatric care registered nurses (*N* = 120; *n* = 69 somatic, *n* = 51 psychiatric)	Schizophrenia/Psychiatric Clinic & Somatic Clinic in a University Hospital	Swedish version of the Attitudes to Persons with Mental Illness Questionnaire (CAMI)	Schizophrenia ranked among the top three conditions with the most negative attitudes, viewed as highly dangerous, unpredictable, socially different, and challenging to recover from, with 47% pessimistic about recovery.
Burns et al., 2000 (47) (United Kingdom, [UK])	GPs and MHPs (*N* = 119; *n* = 52 GPs; *n* = 58 MHPs [29 CPNs; 29 psychiatrists])	Long-term Psychotic Disorders/Primary Care	Author-designed questionnaire regarding difficulty of working with patients and rewards of working with patients	GPs viewed patients with chronic psychosis as burdensome, crisis-prone, and challenging to communicate with, while MHPs were less pessimistic about prognosis and found them more rewarding to care for.
Caldwell & Jorm, 2001 (48) (Australia)	Mental health nurses, psychologists, psychiatrists, general practitioners (*N* = 1508; *n* = 328 mental health nurses; *n* = 535 psychiatrists; *n* = 211 psychologists; *n* = 434 GPs)	Schizophrenia/Multiple settings (i.e., country wide survey)	*Author-designed questionnaire assessing what is wrong, helpfulness of various treatments, likelihood of discrimination, and likelihood of various social milestones	Most mental health nurses believed schizophrenia and depression faced discrimination, with schizophrenia seen as more likely. Providers expected more discrimination against schizophrenia than the public, and negative outcome beliefs correlated with perceived discrimination.
Chang et al., 2014 (49) (United States of America [USA])	Staff of an urban inpatient psychiatric unit (*N* = 29)	Psychosis/Psychiatric Inpatient Unit	Attitudes Toward Working with People with Psychosis	Many trainees felt patients were too ill, citing violence, disorganization, and delusions as obstacles, with short stays limiting psychosocial treatment success.
Chowdhury et al., 2000 (50) (India)	Providers in rural India (homeopaths, local non-licensed practitioners, one Hakim, paramedics) (*N* = 17)	Psychosis/Community health providers	*Bengali version of the Explanatory Model Interview Catalogue (EMIC)	Providers ranked deliberate self-harm as the most stigmatized condition. Providers ranked psychosis second, emphasizing marriage-related stigma and concealing diagnoses, while neither group viewed mental disorders as highly dangerous to others.
Corrigan et al., 2014 (51) (USA)	Nurses, physicians, and psychologists from mental health and primary care clinics (*N* = 166; 42.2% primary care, 57.8% mental health)	Schizophrenia/Veterans Affairs Mental Health & Primary Care Clinics	*A 9-item semantic differential scale of mental health attitudes	Mental health clinic providers were more comfortable with personal mental health care, showing less stigma and greater belief in treatment adherence, which correlated with increased referrals and prescriptions.
Dabby et al., 2015 (52) (Canada)	Residents in psychiatry and psychiatrists (*N* = 103; *n* = 68 psychiatrists, *n* = 35 residents)	Schizophrenia/Multiple settings (i.e., University Psychiatry Department & National Psychiatric Members)	*SDS; Opening Minds Scale for Health Care Providers (OMS-HC); Implicit Association Test	No significant differences in stigma measures between residents and psychiatrists; both groups showed greater social distance toward schizophrenia compared to diabetes mellitus, despite no negative implicit associations and slightly lower-than-average OMS-HC stigma scores.
da Silva et al., 2021 (53) (Brazil)	Psychiatrists attending the 33^rd^ Brazilian Congress of Psychiatry (*N* = 779)	Schizophrenia/Multiple settings (i.e., Country Wide Conference)	Questionnaire with items in 4 groups: restrictions on civil rights, stereotyping, perceived prejudice, social distance	Over half of psychiatrists in the schizophrenia group had high stigma scores, associated with longer time since graduation, higher anxiety, and lower positive affect.
Elizur et al., 1986 (54) (Israel)	Medical students during their 5th year of medical school (*N* = 118)	Acute Psychotic Disorder/University School of Medicine	*Questionnaire assessing Empathic Emotional Tendency, Control Authoritarian and Interpersonal, and Emotional Distance	Conduct disorder received the most negative attitudes, followed by psychosis, which was viewed with a more authoritarian medical approach and frequently diagnosed as mental illness. Students favored psychiatric referral for psychosis.
Fekih-Romdhane et al., 2022 (55) (Tunisia)	Family medicine residents (FMR) and non-medical 3rd year students (NMS) (*N* = 374; *n* = 186 FMR; *n* = 188 NMS)	Schizophrenia/Medical Schools	18-item attitudes questionnaire with four factors: social distance, belief of dangerousness, underestimation of patients’ abilities, and skepticism regarding treatment	Residents were less likely to underestimate patients’ abilities but held more negative attitudes regarding patients’ comprehension of their illness and treatment. Both groups had similar beliefs about social distance, dangerousness, and treatment skepticism.
Fontesse et al., 2021 (56) (Belgium, France, and Canada)	Nurses from Belgium, France, and Canada *(N* = 336)	Schizophrenia/Hospitals, clinical centers, and nurses’ associations	23-item scale of Personal and Perceived Public Stigma (PPPS), an author-designed 22-item of dehumanization, & an author-designed quality of contact scale	Nurses stigmatized individuals with severe alcohol use disorder more than those with schizophrenia, but no significant differences were found in dehumanization between the two groups.
Fujii et al., 2021 (57) (Japan)	Community pharmacists (*N* = 115)	Schizophrenia/Community pharmacies	Stigma Scale towards Schizophrenia for Community Pharmacists (SSCP)	At baseline, pharmacists demonstrated relatively positive attitudes toward patients with schizophrenia, with stigma scores below the midpoint.
García-Carpintero Blas et al., 2024 (58) (Spain)	Undergraduate nursing students (*N* = 217)	Schizophrenia/Public university school of nursing	Attribution Questionnaire (AQ) 27-E9 (Spanish version)	Students showed higher scores on pity and help dimensions, and lower on responsibility and segregation. Being male was associated with lower emotional responses, particularly pity. No effect was observed for previous contact.
Gonzales et al., 2021 (59) (USA)	Medical students, psychology trainees, and licensed clinical psychologists (*N* = 344; *n* = 86 medical students, *n* = 67 clinical psychology trainees, *n* = 93 clinical psychologists)	Schizophrenia/Multiple settings (i.e., snowball sampling)	* AQ; Reported and Intended Behavior Scale; Mental Illness Microaggression Scale-Perpetrator Version (MIMS-P)	Medical students exhibited the highest stigma scores across measures, including negative attitudes, stereotypes, and desired social distance, compared to psychology trainees and psychologists. Prior experience with psychiatric populations was associated with significantly lower stigma levels.
Grausgruber et al., 2007 (60) (Austria)	Non-medical MHPs (psychiatric nurses, social workers, psychologists, physiotherapists and occupational therapists) (*N* = 460)	Schizophrenia/Country wide survey	*SDS	Approximately 25% of staff perceive individuals with schizophrenia as dangerous. Respondents were most accepting of schizophrenia patients as neighbors and least willing to have them care for children, with employment and family acceptance ranked similarly. Staff demonstrated notably more acceptance and belief in recovery compared to the general public.
Hansson et al., 2013 (61) (Sweden)	MHPs in Sweden (*N* = 140)	Psychosis/Outpatient & Inpatient Mental Health Services	12-item Perceived Devaluation & Discrimination Questionnaire	Staff primarily caring for psychosis patients held more negative attitudes regarding trustworthiness, employability, and the seriousness of opinions of former patients, with higher overall stigma scale scores compared to other staff.
Hemingway et al., 2024 (62) (USA)	Resident physicians in Emergency Medicine, Family Medicine, Internal Medicine, Psychiatry, and Transitional Year programs (*N* = 58)	Schizophrenia/Hospital training system	*Attitudes Toward Mental Illness Questionnaire (AMIQ)	Schizophrenia elicited the second most negative attitudes, following multiple inpatient admissions.
Hsiao et al., 2019 (63) (Taiwan)	Mental health nurses from psychiatric inpatient units in Taiwan (*N* = 121)	Schizophrenia/Acute psychiatric wards & Psychiatric rehabilitation wards	*Chinese version of AMIQ	Mental health nurses in this study overall demonstrated negative attitudes toward schizophrenia.
Ishige & Hayashi, 2005 (64) (Japan)	Participants of mental health lectures at the Tokyo Psychiatric Institute (*N* = 786; *n* = 261 psychiatric nurses, *n* = 83 public health nurses, *n* = 76 non-psychiatric care workers, *n* = 75 local welfare commissioners [LWC], *n* = 58 professional probation officers [PPO] and *n* = 229 other workers [non-care workers]).	Schizophrenia/Community lecture series open to multiple occupational groups	The evaluation scale and the modified Social Rejection Scale (m-SRS)	Public health and psychiatric nurses showed higher affective acceptance compared to other occupational groups, though psychiatric nurses demonstrated lower social acceptance, similar to non-care workers. LWCs exhibited relatively high social acceptance but lower affective acceptance, while non-psychiatric care workers and PPOs had lower attitude scores than non-care workers, despite psychiatric education and contact experience.
Jorm et al., 1999 (65) (Australia)	GPs, psychiatrists and clinical psychologists (*N* = 2454; *n* = 1128 psychiatrists, *n* = 454 clinical psychologists, *n* = 872 GPs)	Schizophrenia/Country wide survey	*Author-developed questionnaires regarding the diagnosis of the person described, the helpfulness of various interventions, prognosis with and without professional help, the person’s long-term functioning in various social roles compared to other people, and the likelihood of the person being discriminated against.	Clinical psychologists rated positive outcomes for schizophrenia as more likely and negative outcomes as less likely than psychiatrists, with GPs also rating positive outcomes as more likely than psychiatrists. All groups agreed that discrimination was more likely for schizophrenia than for depression.
Kaitz et al., 2022 (66) (USA)	Mental health providers in the Veterans Health Administration (*N* = 77; *n* = 6 psychiatrists, *n* = 36 psychologists, *n* = 13 social workers, *n* = 16 nurses, and *n* = 6 providers in other disciplines)	Schizophrenia/Midwestern Veteran Affairs Healthcare System	24-item semantic differential scale	Significant differences in stigma scores between male and female providers were found for schizophrenia, with male providers having higher rate of stigma.
Kataoka et al., 2024 (67) (Japan)	Healthcare providers (*N* = 387; *n* = 10 psychiatrists; *n*= 16 psychiatric staff; *n* = 26 physicians; *n* = 237 medical students; *n* = 98 non-medical workers)	Schizophrenia/Multiple settings (i.e., Medical students within one university, healthcare providers via widely distributed online survey)	Author designed 18-item questionnaire for evaluating attitudes toward schizophrenia	Psychiatrists showed the least stigma; medical students, physicians, and non-medical workers had similarly high stigma. Psychiatric education and training were not linked to reduced stigma. Students aspiring to psychiatry had more positive attitudes; female students showed less treatment skepticism. Personal contact had no effect.
Kermode et al., 2009 (68) (India)	Village health workers in rural India (*N* = 60)	Psychosis/Community health providers	*Author-designed questionnaire adapted from mental health literacy survey that included open and closed responses on problem perceptions, causes, helpfulness of treatments and providers, prognosis, and attitudes (partly via social distance measures)	Greater social distance was desired for the psychosis vignette, labeling the issue as depression or a psychological problem, perceiving it as personal weakness, or attributing it to causes like addiction, bereavement, or childhood difficulties.
Khandel Wal et al., 1987 (69) (Ethiopia)	Undergraduate medical students (*N* = 100)	Acute psychosis & Schizophrenia/University Department of Psychiatry	*Author-designed structured questions regarding the presence of an illness and it’s social consequence	Acute psychosis and schizophrenia were perceived as very serious conditions with the worst marital, family, and occupational outcomes, while epilepsy and mental retardation were also viewed as serious but with more favorable social consequences. Alcohol dependence was seen as less serious but with poor prognosis and unfavorable social outcomes.
Korszun et al., 2012 (69) (UK)	Medical students (N = 760)	Long-standing auditory hallucinations and paranoid delusions/Medical Schools Nation Wide	Medical Condition Regard Scale (MCRS)	Students showed the highest regard for patients with pneumonia and the lowest for those with intravenous drug use, with patients with psychosis falling in the middle. Attitudes toward pneumonia became more positive over time, while attitudes toward depression, psychosis, and drug use remained unchanged.
Lawrie et al., 1996 (70) (UK)	Primary care providers (*N* = 71)	Schizophrenia/Primary care registry	*Author-designed 15 attitudinal statements	Primary care providers were less willing to have the schizophrenia vignette patient on their caseload, more likely to refer them to a specialist, view them as violent, and provide general health advice.
Lee et al., 2016 (71) (China)	Health care providers in China (*N* = 50)	Schizophrenia, Attenuated psychosis syndrome (APS), PLEs/Multiple settings (i.e., recruitment through public awareness campaign and e-mail network)	*Seven-domain stigma scale (SDSS)	Participants with mental health work or volunteer experience had significantly lower stigma scores across all vignettes. For the attenuated psychosis vignette, the general public scored higher than healthcare providers on total stigma, social distance, negative affect, and dangerousness, though providers showed no difference from the public in total stigma scores for schizophrenia or psychosis-like experiences.
Magliano et al., 2020 (72) (Italy)	GPs and non-psychiatric medical doctors (MDs) specialists in outpatient community clinics (*N* = 305, *n* = 192 GPs, *n* = 113 specialized MDs)	Schizophrenia/Outpatient community centers	*The revised version of the Opinion Questionnaire	Only 15% fully believed that stopping medication makes individuals with schizophrenia dangerous, while 29.1% fully believed recovery is possible. Those endorsing biogenetic causes were more pessimistic about recovery, more confident in medication efficacy, and more convinced of the need for lifelong pharmacological treatment. Additionally, 75% partially or fully believed lifelong psychotropic drugs are necessary, and 63.9% partially or fully believed stopping medication increases dangerousness.
Mannarini et al., 2020 (73) (Italy)	MHPs (*N* = 102; 57.8% psychiatric nurses, 16.7% psychiatrists, 14.4% psychologists, 5.5% social workers and 5.5% educators)	Schizophrenia/Multiple settings (i.e., regional recruitment)	*CAMI, the Questionnaire on the Opinions about Mental Illness, AQ-27, the Mental Disorders Causal Beliefs (MDCB) scale, and the Mental Disorder Therapy Relationship scale	Providers exhibited high levels of stigmatizing attitudes, particularly regarding dangerousness, social distance, and public avoidance. They showed the strongest preference for distance from individuals with schizophrenia compared to relatives, patients, and students.
Mittal et al., 2016 (74) (USA)	Healthcare providers (*N* = 351 health care providers; *n* = 67 mental health nurses, *n* = 62 psychiatrists, *n* = 76 psychologists, *n* = 91 primary care nurses, *n* = 55 physicians)	Schizophrenia/Veteran Affairs medical centers in the southeast and southcentral USA	*Stereotyping Scale, AQ-9, and SDS	Providers with higher professional contact had lower stigmatizing attitudes and beliefs, which correlated with higher expectations for patients with schizophrenia. Professional contact also had a significant direct relationship with provider expectations, unlike personal contact, which influenced expectations only indirectly through reduced stigma.
Noblett et al., 2015 (75) (UK)	General hospital doctors (*N* = 52)	Schizophrenia/General medical & surgical wards in 3 hospitals	*AMIQ	Negative attitudes were strongest for schizophrenia, personality disorder, and heroin addiction, equating them with attitudes toward ‘criminals.’ These patients were seen as unpredictable and their motives for seeking care viewed with suspicion, with no significant differences in attitudes among these groups.
Oliveira et al., 2020 (76) (Portugal)	Medical students and medical providers (*N* = 353, *n* = 203 students, *n* = 121 non-psychiatry doctors, *n* = 29 psychiatry specialists)	Schizophrenia/Public hospitals	*Portuguese version of AQ-27	Psychiatrists exhibited lower stigmatizing attitudes across all categories except pity, with significantly less fear, anger, and danger perceptions. Both psychiatrists and students demonstrated fewer stigmatizing attitudes in help and avoidance dimensions compared to non-psychiatrist doctors.
O’Reilly et al., 2010 (77) (Australia)	Pharmacists (*N* = 391)	Schizophrenia/Pharmacies	*Author-designed questionnaires regarding helpfulness of different interventions, likelihood of long-term outcomes with and without treatment, and likelihood of long-term functioning	Most pharmacists believed community discrimination was highly likely (61% for depression, 89% for schizophrenia) and rated poor long-term prognosis without professional help. Pharmacists were less likely than other professions to associate schizophrenia and depression with negative outcomes like violence or drug use.
Reavley et al., 2014 (23) (Australia)	Medical providers (*N* =1536; *n* = 518 GPs, *n* = 506 psychiatrists, *n* = 498 psychologists, *n* = 14 no profession indicated)	Early schizophrenia & Chronic schizophrenia Multiple settings (i.e., Country wide survey)	*9-item personal stigma scale, 9-item perceived-stigma scale, & 5-item SDS	Providers perceived others as having highly stigmatizing attitudes toward individuals with schizophrenia, a pattern reflected in personal dangerousness/unpredictability scores, though average personal attitudes were lower.
Richards et al., 2024 (78) (USA)	Undergraduate nursing students (*N* = 110)	Schizophrenia/Undergraduate University	The Prejudice toward People with Mental Illness, Shortened Version (PPMI-SV); Prejudice toward People with Depression, Shortened Version (PPD-SV); and Prejudice toward People with Schizophrenia, Shortened Version (PPS-SV)	Attitudes toward recovery showed strong negative correlations with prejudice toward general mental illness and depression, and a moderate negative correlation with prejudice toward schizophrenia. Additionally, all three prejudice measures were strongly positively correlated.
Servais et al., 2007 (79) (USA)	Clinical psychologists (*N* = 306)	Schizophrenia/Multiple settings (i.e., APA membership recruitment)	6-item semantic differential scale	The target ‘a person with schizophrenia’ was rated as the least effective, least understandable, and second to least safe and desirable (after borderline features), and the most dissimilar to respondents. Psychologists showed extreme ratings, with 69% viewing individuals with schizophrenia as highly dissimilar compared to only 4% for ‘a member of the public.’ The borderline target was rated the least desirable, while the schizophrenia target was rated the most dissimilar.
Strauser et al., 2009 (80) (USA)	Rehabilitation service providers (*N* = 98; *n* = 46 bachelor’s level providers*, n* = 52 master’s level providers)	Psychosis/Multiple settings (i.e., multi-state recruitment)	Psychiatric Disabilities AQ (85)	Providers with a master’s degree reported higher levels of controllability for psychosis compared to those with a bachelor’s degree, indicating greater negative attribution and a tendency to view individuals with psychosis as responsible for their condition. Practitioners with a master’s degree also demonstrated higher stigma for psychosis and cocaine addiction regarding stability and controllability, with psychosis, cocaine addiction, and mental retardation eliciting more stigma than cancer.
Tay et al., 2025 (81) (Singapore)	Allied health staff (*N* = 180; *n* = 11 occupational therapists, *n* = 12 medical social workers, *n* = 23 case managers, *n* = 59 nurse, *n* = 23 physician, *n* = 12 psychiatrist, *n* = 23 psychiatry resident)	Schizophrenia/Specialized mental health care facility	SDS; 9-item personal stigma scale; empathic concern subscale of the Interpersonal Reactivity Index; modified attitudes toward people with schizophrenia scale	Higher education, longer work experience, and having a loved one with mental illness were associated with better attitudes and lower stigma. Being a psychiatrist or psychiatry resident was associated with showed lower stigma; nurses and case managers associated with higher stigma or social distance. No effects of age; female sex linked to greater empathy only.
Tsoi et al., 2021 (82) (China)	Medical students (*N* = 72)	Psychosis/Department of Psychiatry	*Clinician Attitudes Scales version 4 (MICA), Reported and Intended Behaviour Scale (RIBS), Emotional Reactions to Mental Illness Scale (ERMIS)	At baseline, medical students demonstrated moderate social distance, stigmatizing attitudes, and significant fear and anger toward individuals with psychosis, highlighting prevalent stigma prior to intervention.
Valery et al., 2023 (19) (France)	MHPs (*N* = 357; *n* = 147 nurses, *n* = 74 psychiatrists, *n* = 78 psychologists, *n* = 58 others)	Schizophrenia/Multiple settings (i.e., social networks & professional directory recruitment)	STIGMAPRO scale	Theoretical beliefs were the strongest predictors of schizophrenia stigma among MHPs, with biological and similarity beliefs predicting less stigma and categorical beliefs predicting more. Recovery-oriented practices, professional utility beliefs, multidisciplinary team practice, and the psychologist profession were associated with reduced stigma. Independent practice predicted higher stereotypes and social distance, linked to fewer continuum beliefs and perceived similarities. Socio-demographic characteristics like age and familiarity were weak predictors, while personal familiarity reduced stereotypes but work contact frequency showed no association. Multidisciplinary teams promoted less stigma through recovery-oriented practices and increased perceived similarity.
Volmer et al., 2008 (83) (Estonia)	Undergraduate pharmacy students (*N* = 157)	Schizophrenia/Undergraduate pharmacy program	International Pharmacy Students’ Health Survey (IPSHS), SDS, and the 14-item Leeds Attitudes Toward Concordance (LATCon) scale.	Students expressed high social distance from individuals previously hospitalized with schizophrenia, with 87.9% unwilling to share a flat and 98.4% unwilling to have them as a babysitter. Most viewed people with schizophrenia as unpredictable (68.4%) and dangerous (47.5%). However, 96.1% prioritized establishing a therapeutic alliance, and 51% saw consultations as negotiations between equals. Few students attributed barriers to medication counseling to pharmacists’ attitudes and beliefs.
Yashikhina et al., 2022 (84) (Russia)	Psychiatrists (PSY) and nonpsychiatric healthcare providers (NPHP) (*N* = 141; *n* = 20 PSY; *n* = 121 NPHP)	Schizophrenia/State healthcare institutions	7-point Bogardus Social Distance Scale (BSDS) in a version as translated and adapted for the Russian-speaking population	Total BSDS scores indicated greater social distance toward patients with drug addiction and alcohol use disorder, with the lowest scores for depression and epilepsy. Schizophrenia fell between these extremes. PSY and NPHP participants showed similar social distancing tendencies, despite PSY having more knowledge and experience with mental disorders.

Studies marked with * used a patient vignette as part of the stigma measure.

AMIQ, Attitudes Toward Mental Illness Questionnaire; AQ, Attribution Questionnaire; ARMS, At-Risk Mental State; BSC, Bachelor of Science; BSDS, Bogardus Social Distance Scale; CAMI, Community Attitudes to the Mentally Ill; EMIC, Explanatory Model Interview Catalogue; ERMIS, Emotional Reactions to Mental Illness Scale; FMR, Family Medicine Residents; IPSHS, International Pharmacy Students’ Health Survey; LWC, Local Welfare Commissioner; MCRS, Medical Condition Regard Scale; MD, Medical Doctor; MDCB, Mental Disorders Causal Beliefs; MICA, Mental Illness Clinicians’ Attitudes Scale; MIMS-P, Mental Illness Microaggression Scale-Perpetrator Version; m-SRS, modified Social Rejection Scale; NMS, Non-Medical Students; NPHP, Non-Psychiatric Healthcare Providers; OMS-HC, Opening Minds Scale for Health Care Providers; PLEs, Psychotic-Like Experiences; PPD-SV, Prejudice toward People with Depression, Shortened Version; PPMI-SV, Prejudice toward People with Mental Illness, Shortened Version; PPO, Professional Probation Officer; PPPS, Personal and Perceived Public Stigma; PPS-SV, Prejudice toward People with Schizophrenia, Shortened Version; PSY, Psychiatrist(s); RIBS, Reported and Intended Behavior Scale; SDS, Social Distance Scale; SDSS, Seven-Domain Stigma Scale; SSCP, Stigma Scale towards Schizophrenia for Community Pharmacists; STIGMAPRO, Authored developed stigma scale; UK, United Kingdom; USA, United States.

A wide variety of measurements were used to assess stigma among healthcare providers, with a mix of validated questionnaires, vignette and scenario-based assessments, semantic differential scales, specialized tools (e.g., Stigma Scale Toward Schizophrenia for Community Pharmacists [SSCP]), and other methods such as author-designed questionnaires. In total, 41 different measures were used, with 26 of those used only one time. The most frequently used measures were Social Distance Scales (*n* = 8, 18%), Attribution Questionnaires (AQ; *n* = 6, 13%), and Semantic Differential Scales (*n* = 3, 7%) (**Table 1**). The social distance scale measures the level of social distance individuals wish to maintain from a certain population, assessing willingness to engage in various social situations with these individuals (e.g., “How willing would you be to work closely with a person who has schizophrenia?” Not willing at all – Very willing). The AQ evaluates beliefs and attitudes that people have towards mental illness by assessing emotional reactions and discriminatory responses to a provided vignette (e.g., “How dangerous would you feel Harry is?” Not at all – Very much). The semantic differential scales assess attitudes towards individuals with mental illness by measuring participants’ perceptions along a range of bipolar adjective pairs (e.g., dangerous-safe and unpredictable-predictable). The majority of the studies included a patient vignette in their design (*n* = 24, 55%). However, there was considerable variation in content of the vignettes. Twenty-nine percent provided no description of the vignette used, 16% described the individual as functionally well with well controlled symptoms, 25% specify no substance use, 38% use a male vignette, and approximately 20% contain content that may indicate risk of harm. The majority of studies focused specifically on schizophrenia (*n* = 31, 70%) or psychosis (*n* = 7, 16%). One study focused on both schizophrenia and psychosis, while the remaining studies examined related conditions such as general psychotic disorders (*n* = 2), long-standing hallucinations and delusions (*n* = 1), or multiple illness stages including psychotic-like-experiences and attenuated psychosis (*n* = 2; **Table 1**). Additionally, recovery-related beliefs are frequently assessed in the literature as an indicator of stigma, with assumptions that pessimism about recovery reflects therapeutic nihilism or discriminatory attitudes. Several of the studies reviewed here take this approach. However, studies varied in how recovery-related beliefs were assessed, and many did not define “recovery” within their measures. As such, provider responses may reflect different understandings of recovery, ranging from full symptom remission to functional improvement or quality of life, limiting the interpretability of these items as straightforward indicators of stigma. This issue is further discussed in the *Stigma or Realism* section of the Discussion. Lastly, a small number of studies assessed *perceived public stigma*, that is, how providers believe the general public views individuals with SSDs, rather than their own attitudes. While this may help reduce social desirability bias, it does not directly measure providers’ personal beliefs or implicit biases, which limits interpretability. Findings from these studies are noted as such in the text (e.g., as measuring public stigma) to distinguish them from studies assessing personal attitudes.

#### Healthcare providers included

The review encompassed a wide spectrum of healthcare providers, categorized into six main groups: (1) medical students and trainees, including undergraduate and graduate medical students, (2) nurses, including mental health nurses, primary care nurses, and nursing students, (3) physicians, including general practitioners, psychiatrists, and specialists from various fields, (4) psychologists and mental health professionals, including clinical psychologists and other mental health workers, (5) community health workers, including village health workers and community pharmacists, (6) mixed groups, including studies that reported results combining provider types.

These categories were selected to support coherent synthesis and reflect meaningful distinctions in training, credentialing, and general proximity to mental health care. (1) *Medical students and trainees* were grouped separately to capture early-career attitudes and the influence of training stage. (2) *Nurses* were grouped together due to shared credentialing pathways and overlapping caregiving responsibilities across care settings. (3) *Physicians* were grouped based on common medical training, licensure routes, and clinical authority in diagnosis and treatment. (4) *Psychologists and other mental health professionals* were grouped due to their shared focus on behavioral and emotional functioning and their central role in mental health care delivery. (5) *Community health workers*, such as village health workers and pharmacists, were combined given their common roles in frontline service provision and community-based care. (6) *Mixed groups* were used when studies reported aggregated results across provider types that could not be meaningfully separated. We acknowledge that within-group variability exists, particularly among nurses and physicians, whose roles and degree of contact with individuals with psychotic disorders can vary substantially, but grouping was retained to ensure consistency and feasibility in synthesis.

Relatedly, we note that while psychologists are frequently included in stigma research, many studies with psychologist samples focus broadly on “mental illness” or “serious mental illness” rather than schizophrenia spectrum disorders specifically. Because the current review focused only on studies that directly assessed stigma toward SSDs, studies that did not disaggregate findings by diagnosis were excluded. As a result, the body of evidence specific to psychologists’ attitudes toward SSDs is more limited than for other provider groups.

### Stigma by provider type

#### Medical students

Several studies demonstrated unfavorable attitudes toward SSDs within medical students; large proportions of undergraduate medical students believed individuals with schizophrenia could not marry, live at home, or work (86) and others reported high levels of fear and anger (82) and moderate levels of fear and pity (76). Psychotic disorders also received the second most negative attitudinal ratings (following conduct disorder) when compared to three other disorders, were met with authoritarian treatment approaches, and were attributed to social problems or malingering by a minority of students (54). In another sample, individuals with psychosis were rated more favorably than those with intravenous drug use or unexplained abdominal complaints but less favorably than those with pneumonia or depression (69). Beyond attitudinal stigma, medical students endorsed significantly more stereotyped beliefs, negative attitudes, and microaggressive behaviors than psychology students, licensed psychologists, and even the general public, with very large effect sizes observed across all three measures (59).

Notably, medical students consistently reported the highest desired social distance compared to psychology trainees, licensed psychologists, and community members, with very large effects (59). They also showed higher fear and anger compared to psychology trainees and psychiatrists (76) and expressed more pessimism about recovery outcomes (54, 72). Of note, medical students who hoped to become psychiatrists had significantly more positive attitudes than those with other career goals (67).

Overall, these findings align with the intergroup emotion theory and contact hypothesis domains of the integrated model (**Figure 1**), highlighting the role of unfamiliarity and limited patient exposure in shaping emotional and behavioral stigma.

#### Nurses

Nurses’ attitudes toward schizophrenia reveal significant stigma and negative perceptions, with some variation across contexts and nurse types. Compared to non-psychiatric conditions, schizophrenia was viewed as more dangerous, unpredictable, and unusual, with high levels of pessimism about recovery (46, 56). In one study, 47% of nurses in one sample believed that individuals with schizophrenia would never recover—a pessimism in the sample exceeded only by that for dementia (46). However, it is unclear whether participants interpreted “recovery” as full remission, functional improvement, or something else. Somatic nurses (i.e., physical care nurses in a somatic clinic), compared to psychiatric nurses, rated schizophrenia as more dangerous, unpredictable, and harder to talk to, with large (dangerousness and unpredictability) to small effects (hard to talk to) (46). Nurses also demonstrated lower knowledge and favorability ratings towards psychosis compared to other disorders (44). In another sample, higher overall stigma scores were associated with being nurse (81).

Nurses’ attitudes also vary between affective and social dimensions: psychiatric nurses demonstrated relatively high affective acceptance (i.e., emotional warmth, positive feelings, and affective attitude) of patients with schizophrenia but significantly lower social acceptance (i.e., behavioral intentions and desire to keep physical distance), indicating discomfort in real-life scenarios (64). Attitudes further influenced clinical care, with mental health nurses expressing reluctance to involve family in treatment planning for patients with schizophrenia, which negatively impacted the quality of family-centered care (63).

Nursing students that demonstrated more pessimistic views about prognosis had higher rates of prejudice toward individuals with schizophrenia (78), and nurses who rated the likelihood of discrimination from others against schizophrenia as high also expected more negative long-term outcomes (i.e., poor recovery, relapse, chronic impairment) (48). Nursing students in one sample demonstrated high scores in pity and help but low scores on responsibility (i.e., “perception that individuals with MHC can control their symptoms and are accountable for their condition”) and segregation (58).

Broadly, these attitudes reflect attributional and emotional pathways described in **Figure 1**, including perceived dangerousness, fear-based reactions, and pessimism shaped by limited recovery-oriented training.

#### Physicians

Physicians’ attitudes toward schizophrenia varied across disciplines but reveal elements of stigma, pessimism about recovery, and social distance. Among psychiatrists, large proportions endorsed stigmatizing beliefs. In one study, half of the psychiatrists scored in the highest stigma category (53). Another study reported that schizophrenia was associated with moderate stigma among both psychiatrists and non-psychiatrist physicians, with pediatricians showing the highest levels of social distance (84). Psychiatrists and psychiatry residents also reported a large effect of stronger desires for social distance from individuals with schizophrenia compared to a non-psychiatric condition (diabetes), despite the psychotic symptoms being described as well-controlled (52). However, in another study, being a psychiatrist was associated with better attitudes towards individuals with psychotic disorders and lower stigma scores were associated with being a psychiatry resident, compared to medical officers, nurses, medical social workers, case managers, and occupational therapists (81).

Negative attitudes toward schizophrenia were also evident among general practitioners (GPs), who perceived patients with chronic psychosis as burdensome, difficult to communicate with, and difficult to like, and held pessimistic views about prognosis and family burden (47). Likewise, compared to a vignette without schizophrenia, primary care physicians were less willing to manage the schizophrenia patient, more likely to assume they were violent, and more inclined to refer them to specialists, despite the vignette describing symptoms as being well controlled (70). However, one study found an association between greater empathy and being a general medical doctor (81).

Among medical residents, fewer than half endorsed recovery as a possibility, and many expressed attitudes of social distance, beliefs in dangerousness, and skepticism about treatment efficacy (55). Furthermore, schizophrenia elicited the second most negative attitude, following a patient with multiple inpatient admissions, among various patient vignettes (62). Emergency medicine residents demonstrated a medium effect of the most negative attitudes, while residents in their transition year were more positive, though not significantly so.

Physicians’ skepticism regarding schizophrenia recovery was a theme across studies. Fewer than one-third of general practitioners and specialists believed full recovery was possible, while a majority believed psychotropic medications were a lifelong requirement and that stopping medication would lead to dangerousness (of note, the study did not indicate what was meant by “recovery,” which poses limits to the interpretability; 72). Psychiatrists and GPs alike held more negative long-term prognostic (e.g., substance misuse, death by suicide) views for schizophrenia than for depression, with psychiatrists showing the most pessimism (65) Additional stigma was evident in general hospital settings, where patients with schizophrenia were viewed as difficult to engage, untrustworthy, and comparable to criminals due to perceived unpredictability and ulterior motives (75).

#### Psychologists and other MHPs (non-psychiatrist)

Clinical psychologists demonstrated mixed attitudes across studies. In one sample, clinical psychologists made ratings on semantic differential scales of effective–ineffective, understandable–incomprehensible, safe–dangerous, worthy–unworthy, desirable to be with– undesirable to be with, and similar to me–dissimilar to me; individuals with schizophrenia were rated as the least effective, least understandable, and least similar to themselves compared to individuals with depression, borderline features, or the general public (87); effects were large in magnitude. Schizophrenia was rated as the second-to-least safe and second-to-least desirable to be with (after borderline features). Notably, 69% of psychologists viewed individuals with schizophrenia as highly dissimilar to themselves, indicating persistent perceptions of otherness.

However, psychologists displayed more favorable attitudes overall compared to other healthcare providers. Psychology students and licensed psychologists endorsed significantly lower levels of social distance and stigma than medical students and community members, with very large effects (59), and lower stereotyping and discrimination compared to nurses and psychiatrists, with medium effects (19). They also demonstrated greater optimism regarding recovery outcomes compared to psychiatrists (65), although they rated likelihood of discrimination from the public as high.

It is important to note that psychologists and other mental health providers are often analyzed in combined samples, which limits the availability of findings specific to this provider group. Furthermore, many studies with psychologist participant pools focus broadly on mental illness rather than psychosis or schizophrenia. Combined sample findings and comparisons with other provider types are discussed in greater detail in the “Mixed Groups” section.

#### Community health workers

Community health workers had varying degrees of stigma, with some notable differences based on provider type and training background. Non-licensed rural community health workers rated psychosis as highly stigmatized, expressed a stronger desire for social distance from individuals with psychosis compared to those with depression, and associated it with dangerousness, personal weakness, and erratic behavior (50, 68).

Pharmacists and pharmacy students demonstrated similarly complex attitudes. Pharmacists linked schizophrenia with poor prognosis, violence, social and vocational difficulties, and high likelihood of discrimination from others (77). Pharmacy students expressed especially strong stigmatizing attitudes, with high unwillingness for personal interaction and perceptions of dangerousness and unpredictability (83). High social distance was strongly associated with beliefs that people with schizophrenia are difficult to talk to or are to blame for their condition. Conversely, pharmacists in one study demonstrated relatively positive attitudes toward individuals with schizophrenia, with attitudes scoring lower than the midpoint on stigma measures, suggesting less pronounced stigma among this group (57).

#### Mixed groups

##### Collapsing providers

In mixed samples of healthcare providers, stigma toward SSDs varied notably by illness stage, experience, and attitudinal factors.

Regarding illness stage, stigma beliefs and discriminatory behaviors increased progressively from psychotic-like experiences (PLEs) and at-risk mental states (ARMS), and culminated in the highest levels with schizophrenia, with large effects sizes between PLEs and schizophrenia (45). Additionally, while healthcare providers in another sample generally stigmatized schizophrenia and PLEs similarly to the public, they exhibited a large effect of lower stigma toward attenuated psychosis syndrome (71).

Two studies with combined provider groups associated SSDs with higher perceived dangerousness and preference for social distance (73); in one case these beliefs surfaced with a large effect despite the vignette describing the individual as being polite and having no history of substance use (23). However, providers in one sample demonstrated notably greater acceptance, lower social distance, and higher optimism about treatability than the public or patients’ relatives (60).

Certain attitudes correlated with treatment expectations and professional behavior. Agreement with stigmatizing attitudes correlated negatively with expectations of treatment adherence, influencing clinical decisions such as referrals and prescriptions, although no significant differences were observed between professional disciplines regarding these expectations (51). Furthermore, across provider groups, perceived discrimination from others against individuals with schizophrenia was consistently high and strongly correlated with negative outcome expectations, suggesting that beliefs about societal stigma influenced providers’ recovery-related attitudes (48, 65).

##### Comparing providers

Finally, we evaluated stigma in studies that directly compared different healthcare providers. These studies are noteworthy in that they present an opportunity to compare provider views from samples assessed with similar measures. Medical students displayed the highest levels of stigma for negative attitudes and stereotypes with very large effects and the highest scores for social distance compared to psychology trainees, licensed psychologists, and community members (59), although they showed significantly lower stigmatizing attitudes in help, pity, and avoidance compared to non-psychiatrist doctors (76). Similarly, in another sample, medical students demonstrated stigma levels comparable to those of physicians and non-medical workers, with no apparent benefit from psychiatric education or clinical training (67). Psychiatrists displayed lower levels of stigmatizing attitudes than non-psychiatrist doctors and medical students with very large effects (76), lower discrimination and underestimation of patients’ abilities (67), and the lowest levels of fear, anger, and danger (76). However, findings on social distance varied: while one study reported lower social distance preferences among psychiatrists compared to non-psychiatrist doctors (76), another found no significant differences (84). Clinical psychology trainees and licensed psychologists demonstrated significantly lower social distance than medical students and community members (59) and were more optimistic about outcomes compared to psychiatrists and GPs (65). GPs were more likely to view patients with chronic psychosis as burdensome, difficult to communicate with, and prone to creating crises compared to psychologists and psychiatrists (47). Across all groups, there was agreement that individuals with schizophrenia were more likely to face discrimination than those with depression (65).

#### Summary

The results reveal that social distance is the most pronounced feature of stigma among healthcare providers, with medical students displaying the highest levels of unwillingness to engage with individuals with schizophrenia, while psychologists and psychiatrists demonstrate significantly lower levels. Nurses generally associate schizophrenia with higher levels of dangerousness and unpredictability compared to psychiatrists, who show lower levels, and psychologists endorse the lowest level of these beliefs. In terms of recovery beliefs, medical students and general practitioners are more pessimistic, while psychiatrists and psychologists show more optimism. Emotional reactions like fear and anger are highest in medical students, while psychologists and psychiatrists display more empathy and less fear. Nurses, especially psychiatric nurses, express more pity, and their social acceptance is lower. Lastly, perceptions of discrimination from others are strongest in medical students and nurses, and lower in psychologists and psychiatrists. These patterns illustrate how provider role, training, and exposure influence stigma through mechanisms captured across attributional, emotional, and experiential domains of the integrated social-affective model (**Figure 1**).

### Moderators of stigma

#### Professional and contextual moderators

##### Etiological beliefs

Three studies reported on the effects of etiological beliefs. Two studies found associations between biogenetic beliefs and increased stigma related to pessimism about recovery and social distance with a medium effect (68, 72). However, the opposite was also observed: biological etiological beliefs predicted less stigmatization (19). Additionally in that sample, they found that continuum beliefs of schizophrenia were negatively associated with stereotypes and social distance, while categorical beliefs had a positive association.

##### Work setting

Providers working in independent practice reported more stereotypes beliefs and more desire for social distance compared to those working in hospital settings, with a medium effect for stereotyping and a weak effect for social distancing (19). Additionally, they found that belonging to a multidisciplinary team was associated with a lower prejudice, though the effect was weak. Lastly, they found that working in inpatient services did not predict more stigma than working in other settings. However, another sample found that providers working on inpatient psychotic disorders units perceived patients as being “too ill” due to violence, aggression, disorganization, and delusional conviction, as well as stays being too short, as barriers for effective psychosocial treatment interventions (49). Two studies found no effect of work settings on stigmatizing beliefs (48, 51).

##### Years in profession/experience

In one study, providers with more work experience demonstrated fewer stigmatizing attitudes (59), and less stigma regarding dangerousness, unpredictability, and blame, with medium to small effects, respectively. Another study found an association between longer work experience and better overall attitudes towards individuals with schizophrenia but higher stigma scores (81). Providers with higher education exhibited decreased social distance scores in one sample (small effect size; 60) and were associated with better attitudes and lower stigma scores in another sample (81) but had more negative attributions about patients’ responsibility for psychosis compared to providers with less education in another sample (80). Longer time since graduation was positively associated with higher stigma levels (53), and greater years in practice predicted higher stereotypes and social distance, though with weak effects (19). Similarly, doctors in their third year of residency reported significantly higher unfavorable attitudes compared to those in earlier years of residency (55), and fourth year medical students showed greater underestimation of patients’ abilities compared to third years (67). Caldwell and Jorm (48) found no significant effects of experience on discriminatory beliefs.

##### Professional orientation

Recovery-oriented practices and cognitive behavioral therapy theoretical orientations were associated with lower stereotypes and lower prejudices, with medium and weak effects, and recovery-oriented practice was associated with lower desire for social distance, with medium effects (19).

##### Contact

Providers with greater professional contact with individuals with SSDs show lower stigmatizing attitudes, higher expectations of patients with schizophrenia (74), and less negative attitudes, though with weak relationships and small effects (46). Similarly, another study found that patient contact predicted more positive implicit attitudes (52). However, three other studies reported no significant association (19, 58, 65), and one reported that staff who primarily treated individuals with psychosis held more negative attitudes compared to those treating other diagnostic groups (61).

##### Personal familiarity (friends/family/self)

Several studies reported that having friends, family members, or personal experience with mental illness was associated with reduced stigma. Personal familiarity significantly lowered stigmatizing attitudes (76, 81), reduced social distance (81, 83), led to fewer stereotypes (small effect) (19), increased positive attitudes (small effect) (62), and significantly increased regard for patients with schizophrenia (medium effect) (69). Providers’ comfort with seeking mental healthcare for themselves was weakly associated with reduced stigmatizing beliefs and more optimistic treatment expectations (51). However, three studies found no association (60, 67, 68). Additionally, one study reported that while higher personal contact with mental illness had a moderately large negative association with stigmatizing attitudes and beliefs, the direct relationship between personal exposure and provider expectations was not significant (74).

#### Demographic moderators

##### Age

In several studies, older providers exhibited higher stigma levels. Specifically, older age was associated with increased pity (76), less socially accepting attitudes (64), and greater stereotyping, although this was a small effect (19). In contrast, other studies found reduced stigma among older participants, with less negative attitudes about dangerousness and unpredictability (46), and negative correlations with social distance scores (55), though the effects were small in both. However, some studies found no effect of age (60, 68, 81) or mixed results, with one study finding no effect for prejudice and social distancing in spite of the greater stereotyping (19). Younger general practitioners, on the other hand, rated negative outcomes (e.g., substance misuse, death by suicide) for schizophrenia more highly than older counterparts in one study (65).

##### Sex

Multiple studies identified higher levels of stigma among male providers compared to female providers, including more negative attitudes (59), more stigmatizing beliefs (55, 66), and pessimism for recovery, with small to medium effects (46). One study found higher stigma scores among male providers, but the results were not statistically significant (50). Results for female providers were mixed; with one study finding that female medical students had significantly higher levels of regard for patients with schizophrenia, though the effect was small (69), less underestimation of patients’ abilities (67), greater empathy (81), and more positive attitudes toward schizophrenia than male providers, though not statistically significant (75). Conversely, some studies reported that female providers rated negative outcomes as more likely (65) had higher levels of skepticism regarding treatment (55), and had higher emotional responses for fear, anger and pity (58). Numerous studies found no effects of sex across stigma components: socially accepting attitudes (64, 81), social distance scores (68, 81), and discriminatory beliefs (48). Additionally, two studies found higher stigma amongst men for other diagnoses as well, including PTSD, eating disorders, and depression (46, 66). This is in line with previous literature finding higher stigma in men for mental illness overall (88, 89).

## Discussion

This systematic review synthesized findings from 44 studies examining stigma toward SSDs among healthcare providers. Overall, findings indicate that stigma towards individuals with SSDs is more pronounced among general practitioners, community health workers, and medical students—particularly those with limited psychiatric training or exposure. In contrast, psychiatrists, psychologists, and providers with specialized training in mental health reported lower levels of stigma. Familiarity with SSDs, through training or patient contact, appears more influential than professional role alone. See **Table 2** for a condensed summary of results.

**Table 2:** Summary of results.

Feature of Stigma	Medical Students	Nurses	Psychiatrists	Psychologists	Community Health Workers	Moderators
Social Distance	Strong association (++)	Moderate, more accepting than med students (++)	Lower social distance (+)	Lowest social distance among provider groups (––)	High for psychosis, lower for others (++)	Familiarity (++), Experience (±)
Dangerousness & Unpredictability	Strong association (+++)	Dangerous but less than students (++)	Lower levels (+)	Lowest association with dangerousness and unpredictability (––)	Similar to med students (++)	Age (±), Gender (±)
Beliefs About Recovery	More pessimistic (+++)	Some optimism, but more pessimistic than psychiatrists (++)	Optimistic, though not consistently most optimistic (–)	Optimistic compared to med students and nurses (––)	Varies by training/setting (±)	Age (±), Orientation (+)
Emotional Reactions	High fear and anger (+++)	Greater pity, moderate fear (++)	Lower fear and anger (+)	Lowest levels of fear and anger; more empathetic (––)	Mixed reactions (±)	Familiarity (++)
Perceptions of Discrimination	Perceive discrimination; may influence expectations (++)	Similar to students, less extreme (++)	Mixed perceptions; less likely than others but still present (±)	Lower perceptions but still acknowledge (±)	Mixed; some perceive more discrimination (±)	Setting (±), Familiarity (++)

Character markings reflect the general strength and direction of findings as synthesized in the results section.

+++ = Strong evidence of high stigma; multiple studies with consistent findings and/or large effects.

++ = Moderate evidence of high stigma.

+ = Limited or weak evidence of high stigma.

± = Mixed or inconsistent findings across studies.

– = Limited or weak evidence of low stigma.

–– = Moderate evidence of low stigma.

Moderator-level symbols follow the same logic, reflecting the strength of association between the moderator and each stigma feature.

The most consistent moderators linked to lower stigma were increased personal and professional contact, along with recovery-oriented frameworks. In contrast, findings for demographic (e.g., age, sex) and professional factors (e.g., years in profession, workplace setting) were mixed across studies. The variability may reflect contextual factors or interactions between moderators, but is also likely driven by methodological differences. Studies varied widely in how stigma was assessed (e.g., attitudes, behaviors, emotions) and in the tools used, including author-developed scales with unclear psychometric properties. This variability makes it difficult to make meaningful, confident comparisons across studies, even when targeting similar constructs or provider types. Vignette content also varied: over a fourth of studies using vignettes did not provide the stimuli, and those that did varied in content (e.g., some having well-controlled symptoms, others including signs of violence or substance use) which was not experimentally manipulated. These methodological inconsistencies highlight the need for more transparent and standardized approaches in future research.

Additionally, variability in moderator findings may reflect intersectionality between factors such as age, sex, workplace setting, and personal experience with mental illness. These factors may influence stigma in complex or even opposing ways; for example, older individuals may hold stigmatizing views due generational norms, but this may be offset by personal exposure to mental illness. Cultural context is another likely contributor to variability, with studies spanning 19 countries. Cultural beliefs shape how mental illness is understood, whether through a moral, supernatural, or biomedical lens (90, 91); these frameworks influence both the expression and measurement of stigma among healthcare providers.

Overall, the findings of this review align with prior research on stigma within healthcare settings, particularly in identifying misconceptions about dangerousness and poor recovery outcomes as pervasive among providers (2, 10, 21). However, this review extends the literature by systematically comparing stigma across multiple provider groups and identifying potential key moderators of stigma, such as sex and personal experience with mental illness.

### Findings in theoretical context

These findings align with the contact hypothesis (22), particularly within the microsystem of provider-patient interactions (32), showing that mental health providers and providers with personal experience tend to exhibit lower stigma. Critically, this suggests that not all contact is equally beneficial—brief, task-focused interactions, more common among general healthcare providers and community health workers, may lack the relational depth needed to challenge stereotypes. In contrast, structured, supervised engagement in mental health settings likely facilitates more meaningful, stigma-reducing contact. Existing contact-based stigma interventions (92–94) may benefit from inclusion of therapeutic practices such as consistent debriefing and supervisory reflection. This may foster more constructive, compassionate, stigma-reducing contact—standard in psychology settings— and may help generalist providers process biases, foster more compassionate care, and reduce stigma.

The results also highlight the relevance of intergroup emotion theory (28) at the microsystem level, particularly in how providers view individuals with SSDs as an outgroup. For example, in one of the included studies, 69% of clinical psychologists (typically providers with the lowest levels of stigma) rated individuals with schizophrenia as highly dissimilar from themselves (87). Such perceived dissimilarity can elicit fear and discomfort, especially among trainees or provider with limited exposure, and these affective reactions often drive avoidance behaviors (95). This suggests a need for interventions that directly target emotional regulation, such as empathy-building exercises or experiential learning exercises.

Causal attributions also emerged as an important mechanism, consistent with attribution theory (25, 80). Although biogenetic explanations may reduce blame, they may simultaneously increase pessimism about recovery and reinforce perceptions of dangerousness, suggesting a dual-edged nature of how such attributions shape emotional and behavioral responses. Such effects have been documented as unintended negative consequences of anti-stigma campaigns that emphasize biogenetic frameworks (96–99). To mitigate this, interventions should promote recovery-oriented narratives alongside psychoeducation that presents a biopsychosocial framework, rather than a purely biogenetic one. These findings collectively support the integrated social-affective model (**Figure 1**), illustrating how stigma emerges from intersecting psychological and institutional forces.

### Implications for practice and intervention

Stigma across provider types was linked to gaps in knowledge and experience with SSDs. This highlights the need for integrating comprehensive mental health training into non-specialist fields such as general medicine, nursing and pharmacy. Early, sustained clinical exposure to SSDs may challenge stereotypes and foster empathy and compassion (82, 100). For practicing providers, continuing education that combines evidence-based content with meaningful patient interactions could be particularly effective (101), especially given recent evidence that private practice providers demonstrate higher levels of stigma (19).

While many interventions target individual providers, systemic change is critical to address structural inequities that perpetuate stigma. This includes policy reforms mandating comprehensive mental health training across professions, and restructuring healthcare systems to improve access and interdisciplinary collaboration. One such model is the United States Veterans Health Administration’s PCMHI program, where integrating behavioral health into primary care supports team-based care and bridges knowledge gaps between primary and general care providers with MHPs (102–104). Workplace policies that reduce burnout and promote inclusivity also create environments conducive to stigma reduction. Drawing on the “Seed and Soil” framework (105) interventions are more effective when implemented in supportive environments with the psychological affordances for change. Reducing systemic barriers such as high workloads, inadequate funding, and limited training opportunities is thus crucial. Notably, providers in multidisciplinary settings report lower stigma (19), suggesting that fostering collaboration and shared responsibility, similar to that of the VHA PCMHI, may simultaneously reduce burnout and improve knowledge and contact (106, 107).

### Stigma or realism?

Identifying and labeling providers’ perceptions as stigma is not always straightforward. In clinical practice, healthcare providers may form negative appraisals of patients with SSDs—that *appear* stigmatizing—based on realistic assessments of the research literature or direct clinical experience, such working with patients with aggression or impaired social functioning, which may be more pronounced in certain contexts (e.g., on psychiatric in-patient units). These appraisals may not stem from bias, but rather from the complex, challenging experiences providers face in managing signs, symptoms, and behaviors associated with a serious and complicated mental disorder. Similarly, they may reflect providers’ awareness of empirical evidence on SSDs; for example, higher rates of socio-occupational functioning are evident throughout the literature (108, 109). If, as is done in a number of the studies included here, a provider endorses the belief that individuals with an SSD may struggle in social relationships, this may reflect an evidence-based appraisal of outcome. Related concerns, such as risk of harm, poor prognostics, or likelihood of recovery, can also emerge from research on SSDs (110, 111). These negative prognostic views may not be pure reflections of stigma, but data-based views shaped by the complex etiology and associated outcomes experienced by individuals with an SSD.

Still, such negative appraisals—even those that *could* be said to be evidence-based—must be critically examined in terms of their clinical application and impact on individuals with SSDs. This is particularly important when such appraisals influence discriminatory and incendiary behaviors by providers. Prior research has demonstrated that provider behaviors influence patient interactions and outcomes, both positively and negatively (112–114). Staff trained in evidence-based interventions, such as social learning principles, show marked improvements in therapeutic interactions and fewer negative behaviors towards patients, which in turn improved patient compliance and social functioning (79, 115). Likewise, expressed emotion (EE) literature shows that staff who express warmth and support, while avoiding criticism or hostility, promote positive relationships and better outcomes for patients (116). Positive staff-patient relationships, where staff attribute patient difficulties to external factors internal causes, further contribute to better patient outcomes (117). Furthermore, when staff adopt a person-centered approach, emphasizing collaboration and empathy, patients demonstrate better engagement and recovery outcomes (118). These findings highlight how provider attitudes and perceptions shape behavior and how behavior, in turn, shapes patient outcomes.

Even evidence-based appraisals can inadvertently reinforce stigma when applied rigidly, especially when they stem from limited exposure to SSDs or reflect a lack of recovery-oriented training. For example, this review identified three instances in which vignettes described individuals with well-controlled symptoms, yet providers consistently expressed a desire for social distance and concerns about potential dangerousness (23, 52, 70). One likely contributor is the clinician’s illusion—provider’s tendency to overestimate overall illness severity due to primarily seeing the most severe, treatment seeking cases (119). In essence, this leads to a skewed perception of SSDs and recovery potential as a whole. Exacerbating this, providers may fall prey to the ecological fallacy: the mistaken assumption that group-level trends necessarily apply to individuals, resulting in clinical judgements that conflate statistical generalities with individual prognosis (120). Stigma-based or not, negative clinician perceptions require further empirical and applied attention.

### Limitations

This review has several important limitations. First, most included studies were conducted in high-income Western countries, limiting the generalizability to non-Western and low- and middle-income countries. Sociocultural differences in stigma and healthcare systems in these underrepresented regions may yield different patterns of provider attitudes. Second, although we did not formally assess risk of bias for each included study, we note substantial methodological heterogeneity across studies, particularly in the use of measurement tools, vignette descriptions, and sample sizes. The heterogeneity in study designs may introduce inconsistencies in how stigma is defined and interpreted across studies. Most studies relied on self-report measures, which are subject to social desirability bias. This likely led to underreporting of stigmatizing attitudes, particularly given the target population is healthcare providers. While some studies attempted to reduce this by measuring perceived bias as a proxy for personal bias, such perceptions may represent realistic appraisals of public attitudes (37) rather than implicit or internalized beliefs. Third, because we only included published studies, there is a risk of publication bias. Moreover, the studies span several decades, with some published as early as the 1980s. While this allowed for a more comprehensive synthesis, it also introduces variability, as societal attitudes toward mental illness and clinical practices have likely changed over time. Findings should be interpreted within their historical context. Finally, although this review followed PRISMA guidelines, the review protocol was not pre-registered.

### Future directions

Several critical gaps remain. The current literature is dominated by cross-sectional study designs; longitudinal studies are needed to assess how provider attitudes evolve over time, particularly in response to training, increased patient contact, or meaningful systemic changes (e.g., updating practice guidelines to emphasize recovery-oriented care, reducing provider caseloads). While individual-level factors like training and exposure have been well-documented, future research should examine how interdisciplinary collaboration and structural reforms contribute to stigma reduction. Mixed method approaches that integrate quantitative and qualitative data can illuminate how providers experiences, beliefs, and institutional contexts shape stigmatizing behaviors.

Additionally, future research should also explore the impact of self-stigma interventions on providers’ attitudes, as this may have the potential to influence both patient outcomes and provider perceptions. At least six established interventions target self-stigma (121), raising the interesting question: if providers work with patients actively working to reduce self-stigma and discussing their experienced stigma, might this increase providers’ awareness of their own biases? Exploring this dyadic dynamic could offer valuable insight into how patient self-empowerment and disclosure may influence provider attitudes, contributing to a reciprocal process of stigma reduction, an unexplored interpersonal feedback loop.

Critically, more work is needed on potential mechanisms of provider stigma. These findings and the broader literature underscore that stigma is multifaceted and context-dependent. Moreover, emerging research suggests that prejudice-reduction strategies in general rarely produce meaningful and lasting effects (122). This raises the need to re-evaluate our targets: focusing on the assessment and change of provider beliefs, explicit or implicit, may be only marginally impactful for real-world change in patient care (122, 123). It is established, here and throughout the literature base, that these beliefs exist across time and among healthcare providers; beliefs are difficult to modify—discriminatory behaviors, instead, may be a more measurable and modifiable target for provider change. Using social marketing principles (122), these interventions can be designed to align with providers’ professional values, emphasizing practical benefits such as improved patient outcomes, enhanced provider efficacy, and fewer workplace barriers to implementing inclusive care.

Addressing provider-based stigma is vital for improving the outcomes of the individuals we serve. Targeted, multi-level interventions hold the potential to transform individual provider attitudes and the broader healthcare systems in which they operate. Reducing stigma among providers is essential for fostering recovery-oriented care and promoting equity for one of the most marginalized patient populations.

## Data Availability

The original contributions presented in the study are included in the article. Further inquiries can be directed to the corresponding author.
